# Editorial: Social science contributions to public health

**DOI:** 10.3389/fpubh.2025.1680022

**Published:** 2025-09-08

**Authors:** Kirrilly Thompson, Tambri Housen, Joanne Talwar

**Affiliations:** ^1^College of Medicine and Public Health, Flinders Health and Medical Research Institute, Flinders University, Adelaide, SA, Australia; ^2^School of Medicine and Public Health, University of Newcastle, University Drive, Callaghan NSW, Callaghan, NSW, Australia; ^3^National Centre for Epidemiology and Population Health, The Australian National University Acton, Canberra, ACT, Australia

**Keywords:** social science and humanities, qualitative social science, One Health, human behavior, behavior change, public understanding of science, collaboration, public health

## Background

Epidemiological studies and medical interventions are essential for understanding and addressing public health issues. However, as conveyed by former Director of the United States Centers for Disease Control and Prevention Bill Foege, “public health is at its best when we see and help others see the faces and the lives behind the numbers” (1). In the social sciences, qualitative research methods are commonly used to gain first-hand insight into human experience, behavior, decision-making and meaning-making. This “insider” knowledge makes it possible to develop strategies and policies that are contextually relevant, responsive to community needs, and inclusive of marginalized populations. There are many examples of qualitative research methods being used to generate evidence to support public health policy decisions, evaluate program effectiveness, and guide resource allocation to promote health equity (2). For this Research Topic, we called for articles demonstrating the ability of social science and qualitative methods to provide an understanding of the complex social dynamics and lived experiences that shape health outcomes.

## Contributions

The six contributions to this Research Topic illustrate the ways in which qualitative social science can be used to understand and therefore address the broad social, cultural, political, religious and/or historical dimensions of a range of public health topics. For instance, Frampton et al. discuss vaccine hesitancy in South Africa to emphasize the importance of “socio-theological influences on engagements with public health interventions” (page 2). Their overview highlights the impact of colonialism and apartheid on responses to public health interventions originating from European and North American research.

Carter et al. describe two case studies where social and behavioral sciences have played an important role in addressing complex public health issues; the global COVID-19 pandemic and the 2021 New South Wales mouse plague in Australia. The integration of qualitative social science methods into public health research is also illustrated by Willemsen et al., who used a mixed methods approach to consider infection prevention and control (IPC) practices in small animal veterinary practices in Australia. The contribution from White et al. outlines the key features of five case studies undertaken by one Local Health District in New South Wales. Each case study illustrates not only how qualitative social science methods and approaches were employed, but how research findings were utilized to inform public health policy at local and national levels. Indeed, the public health gains for managing zoonotic diseases and pandemics can be attributed to effective engagement with qualitative social science.

Together, contributions highlight not only the value of qualitative social science to public health, but the importance of collaboration across disciplines and sectors. This is particularly evident in relation to One Health, which supports a comprehensive public health agenda by highlighting the interdependency of human, animal and environmental health (3, 4). The One Health approach is therefore fundamentally multidisciplinary, uniting specialists within and between academia, government, organizations and communities. Social scientists typically contribute to multidisciplinary One Health research teams by helping understand and influence human behavior and decision making, amongst other things (5). To more fully (and critically) approach One Health as a complex system, Carter et al. highlight the need to include scholars from the humanities as well as the social sciences. They point to ethicists, historians, philosophers, educators and legal scholars who may not be involved in primary data collection but can provide essential insight to the public health impact of policy, governance and institutions.

In Table 1 of their contribution, Carter et al., outline seven contemporary One Health priorities and list relevant examples of capabilities of the social and behavioral sciences and humanities. These contributions can address the broader socio-cultural, historical and political dimensions of One Health issues whilst also facilitating a critical analysis of the production of One Health knowledge and the operation of One Health systems. Their identification of the benefits of involving the full range of the social and behavioral sciences and humanities in One Health is a reminder that social science contributions to public health similarly benefit from collaboration with the humanities. Additionally, Frampton et al. recommend also involving media and communications experts in public health collaborations, specifically regarding pandemics. Levites Strekalova et al. examine the utilization of two U.S. policy tools to highlight the importance of involving experts in health services, management, and policy. The extensive multidisciplinary and multisectoral collaborations described throughout this Research Topic are important not only to respond to major public health events like pandemics, but to reduce risk and build community preparedness.

Nonetheless, high quality and impactful public health outcomes are not inevitable results of multidisciplinary collaborations. In their contribution to this Research Topic, Meyer et al. utilize a qualitative social science approach to understand the experiences of senior-level health promotion researchers involved in a global urban health promotion initiative spanning five cities of low- and middle-income countries. They identify potential points of tension when experts collaborate to resolve complex health promotion issues. Their research suggests that successful collaboration requires clarity, support and guidance.

Whilst “scientific” solutions like medicines and vaccines can make a significant positive impact on public health issues, they are often publicly resisted. The conditions for the acceptance and rejection of medical public health interventions are at least as important as their effectiveness. One of the benefits of involving social scientists in public health and medicine is to continue to challenge the idealistic belief that scientific knowledge should be sufficient for humans to make healthy decisions. For example, in relation to vaccination hesitancy, Frampton et al. eloquently explain:

Those who refuse vaccines can be labeled as simply scientifically “ill-informed.” But most of those who accept vaccines are also largely uninformed about the intricacies of vaccine science, and those who decline or question vaccines can be quite knowledgeable about them. Assuming a lack of understanding as the root cause of vaccine hesitancy fails to do justice to the complexity of human approaches to—and decision-making about—health and our bodies'.

## Final remarks

Whilst most positivistic and experimental scientists are only too aware of the fact that education alone is insufficient to influence behavior, they are often frustrated by an apparent lack of public trust and rational decision making. The social sciences and humanities are uniquely positioned to use qualitative research methods to not only explain how “resistance” makes sense to individuals, but provide insight into how to influence behaviors, attitudes and beliefs that undermine public health outcomes. However, the social sciences and especially the humanities are often considered inferior to medical science and experimental approaches (6)—hence the feasibility of this Research Topic despite a long history of social science research within public health. Until the social sciences and humanities are treated with as much prestige as other sciences and receive resourcing accordingly, developments from the “hard sciences” will fail to meet their full potential for improving public health. We hope that this Research Topic helps not only to illustrate the contributions to public health from social science, qualitative research and the humanities but to normalize their engagement in public health research, practice and extension.
